# Assessment of major ions and trace elements in groundwater supplied to the Monterrey metropolitan area, Nuevo León, Mexico

**DOI:** 10.1007/s10661-017-6096-y

**Published:** 2017-07-15

**Authors:** Abrahan Mora, Jürgen Mahlknecht, Laura Rosales-Lagarde, Arturo Hernández-Antonio

**Affiliations:** 1 Centro del agua para América Latina y el Caribe, Tecnológico de Monterrey, Av. Eugenio Garza Sada Sur N° 2501, 64849 Monterrey, Nuevo León México; 2 Physical Science Department, College of Southern Nevada, 6375 W Charleston Bldv, Las Vegas, NV 89146 USA

**Keywords:** Monterrey metropolitan area, Mexico, Groundwater, Trace elements, Water quality

## Abstract

The Monterrey metropolitan area (MMA) is the third greatest urban area and the second largest economic city of Mexico. More than four million people living in this megacity use groundwater for drinking, industrial and household purposes. Thus, major ion and trace element content were assessed in order to investigate the main hydrochemical properties of groundwater and determine if groundwater of the area poses a threat to the MMA population. Hierarchical cluster analysis using all the groundwater chemical data showed five groups of water. The first two groups were classified as recharge waters (Ca-HCO_3_) coming from the foothills of mountain belts. The third group was also of Ca-HCO_3_ water type flowing through lutites and limestones. Transition zone waters of group four (Ca-HCO_3_-SO_4_) flow through the valley of Monterrey, whereas discharge waters of group 5 (Ca-SO_4_) were found toward the north and northeast of the MMA. Principal component analysis performed in groundwater data indicates four principal components (PCs). PC1 included major ions Si, Co, Se, and Zn, suggesting that these are derived by rock weathering. Other trace elements such as As, Mo, Mn, and U are coupled in PC2 because they show redox-sensitive properties. PC3 indicates that Pb and Cu could be the less mobile elements in groundwater. Although groundwater supplied to MMA showed a high-quality, high mineralized waters of group 5 have NO_3_^−^ concentrations higher than the maximum value proposed by international guidelines and SO_4_^2−^, NO_3_^−^, and total dissolved solid concentrations higher than the maximum levels allowed by the Mexican normative.

## Introduction

The city of Monterrey is located in northeastern Mexico, close to the US border region. With more than four million inhabitants, the Monterrey metropolitan area (MMA) is considered the third greatest metropolitan area and the second largest economic city of Mexico (INEGI 2010). This city hosts most of biggest industries, and it is an important location for the development of commercial and economic activities. The water supplied to MMA comes from groundwater (40%) and surface water (60%). Groundwater originates from several “wellfields” located within or a short distance from urban areas, whereas superficial water sources include three main reservoirs: La Boca dam, Cerro Prieto dam, and El Cuchillo dam (Sisto et al. 2016). Because this important industrial and urban center is situated in a semi-arid region, the scarce rainfall (approximately 600 mm per year) and the high temperatures during most of the year decrease the surface water accessibility around the city and surrounding populations. An example of this is the high water evaporation rate in El Cuchillo dam due to the large water surface and low depth of the reservoir (Masuch et al. 2002). Moreover, due to the rapid population growth (which reached 4.5 million inhabitants in the last years), high urbanization rate, and increased industrialization, more water resources for present and future use are required in the MMA. Owing to the vulnerability of the MMA water supply system has been enhanced because the increased reliance on surface water sources (Sisto et al. 2016), there is a real necessity to increase groundwater abstraction in the city and surrounding locations, especially during dry periods. However, with an increasing demand for groundwater, water quality tends to be deteriorated and the hydrological systems of this zone can be under severe stress almost permanently.

The chemical composition of groundwater plays a key role in assessing the quality of water. In unpolluted systems, major ions in groundwater are provided by weathering of rocks and the quality of water is related to several factors such as geology, weathering regime, quality and quantity of recharge water, and water-rock interaction (Sethy et al. 2016). Nevertheless, rapid industrialization and activities such as agriculture and cattle raising can increase the concentration of several ions and trace elements in groundwater, which results in deterioration of the water quality (Kim et al. 2015; Bardsley et al. 2015). Similarly, the overexploitation of groundwater would lead to loss in the water quality because greater rates of salinity rise may occur around the end of groundwater flow lines (Ajdary and Kazemi 2014).

Owing to large urban areas demanding a large amount of water for drinking, domestic and industrial purposes, studies about the quality of groundwater in megacities such as Shanghai, New Delhi, Tokyo, and Hong Kong have received increased attention (Leung and Jiao 2006; Gao et al. 2012; Thuyet et al. 2016; Singh et al. 2017). Similarly, current published information regarding groundwater quality indicates that both major ion and trace element abundances can be a matter of health concern for humans. For example, high groundwater salinity can increase the risk of high blood pressure or hypertension (Talukder et al. 2017), whereas the scarcity of essential alkaline-earth elements such as calcium and magnesium in drinking water can increase the risk of coronary heart disease mortality in humans (Chao et al. 2016; Jiang et al. 2016). Also, high consumption levels of drinking water nitrate may induce methemoglobinemia or “blue baby syndrome” (Fan and Steinberg 1996), while drinking water containing high sulfate concentrations may produce a laxative effects in humans (WHO 2011).

On the other hand, trace elements can be divided into essential (Cu, Cr, Co, Fe, Mn, and Zn) and non-essential (As, Cd, and Pb) elements. Non-essential trace elements can induce some pathologies in humans such as neurocognitive impairments, cardiovascular diseases, and several kinds of cancer. However, the high levels of essential trace elements in groundwater used for human consumption could also represent a high risk for health (Calderon 2000). Therefore, the assessment of human health risks from exposure to trace metals in groundwater sources supplied to highly populated areas has received increased attention. Studies performed in Pakistan indicate that more than 74 million inhabitants are subjected to health risk from As, Cd, Cr, Fe, Mn, Ni, and Pb contamination of groundwater (Bhowmik et al. 2015). Similarly, other studies carried out in highly populated states of Nigeria (more than 16 million inhabitants) suggest a high risk of cancer development due to the ingestion of groundwater with elevated levels of Cr, Pb, As, and Cd (Ayedun et al. 2015). In addition, studies carried out by the US Geological Survey in public-supply wells distributed across 35 states of the USA indicated that the chemical mixtures in groundwater that had the greatest potential toxicity were primarily composed of trace elements (including As, Sr, and U) (Toccalino et al. 2012). Arsenic has been classified as the principal and most potent hazardous trace element due to its high toxicity and the fact that groundwater can have high levels of As depending on the geological conditions. Elevated As concentrations in groundwater has been associated with skin cancer and a decline in children’s intellectual functions and other neurologic outcomes in several countries of southeastern Asia (mainly in the highly populated Bangladesh) and in some locations of the USA (Wasserman et al. 2014; Muehe and Kappler 2014; Mayer and Goldman 2016).

Studies performed in shallow groundwater of agricultural lands surrounding the MMA have indicated a decrease in the water quality in several areas because total dissolved solid values in groundwater increase gradually in the direction of groundwater flow (Ledesma-Ruiz et al. 2015). Indeed, the primary reactions contributing to salinity are water-rock interactions (including the weathering of salt-rocks and dedolomitization) and dissolution of soil gas carbon dioxide. Similarly, assessments of nitrate sources using a multi-trace approach indicates that animal manure and sewage from septic tanks are the main source of nitrate pollution in groundwater of these agricultural areas (Pastén-Zapata et al. 2014). Although these works refer to the quality of groundwater in several areas close to Monterrey, the quality and chemical composition of groundwater supplied to MMA have not been yet evaluated. Therefore, the objectives of this research were to investigate the main hydrochemical characteristic (major ion and trace element composition) and determine whether groundwater taken from several wells and/or wellfields poses a threat to the MMA population.

## Study area

### General settings

The MMA is the most populated city of the Nuevo León State and the third biggest city of México. The metropolitan area is formed by the autonomous municipality of Monterrey and 11 municipalities of the Nuevo León State, including Santiago. All the urban and industrial area covers a surface of 6.680 km^2^. To the west, it is bordered by mountain ranges composed of carbonated and clastic marine sedimentary rocks, which have an elevation of at least 2100 m above sea level (masl). This mountain belt is known as Sierra Madre Oriental (SMO), which stretches along northeastern and central Mexico. To the east, the elevations decrease to about 400 masl and the city is bordered by the tectonic province of Gulf Coastal Plain, which corresponds to a thick sequence of Tertiary clastic sediments characterized by an extensional deformation (Ortíz-Urbilla and Tolson 2004).

The MMA comprises a semi-arid climate with a mean annual temperature of about 22 °C, with a minimum value of about −10 °C during winter, and a maximum value of about 45 °C in summer. Although the annual mean precipitation is 587 mm, rainfall in the area varies according the topographic elevations, with the flanks of mountains showing the higher values. In fact, the precipitation is determined by warm air masses coming from the Gulf of Mexico, which move across the SMO and thus generating high precipitation values at the mountain flanks. Overall, long-term data obtained between 1941 and 2014 indicates a high inter-annual variability of precipitations, with several prolonged dry periods and other short periods of exceptional precipitations produced by hurricanes (Sisto et al. 2016).

**Fig. 1:**
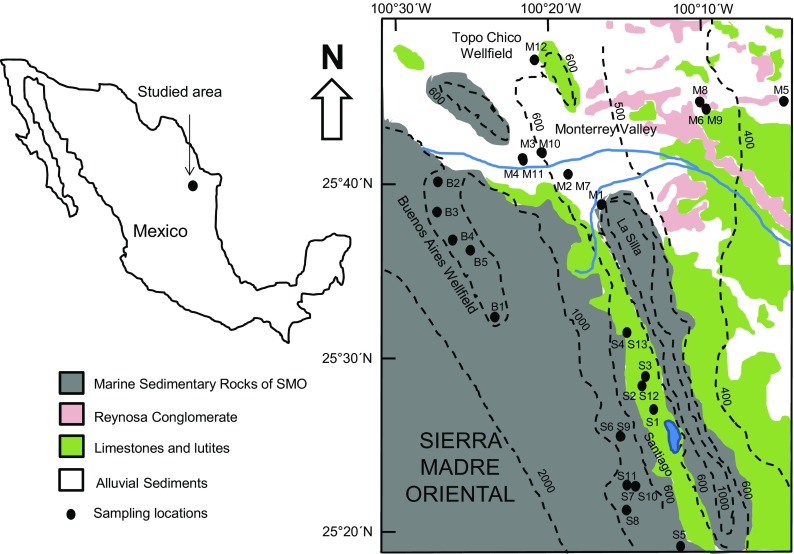
Map of the geology of the Monterrey metropolitan area showing the groundwater sampling locations

According with the topographic path of the study area, surface and groundwater flow from the high ranges of SMO (SW-W) to NE-E direction (Fig. 1). La Silla and Santa Catarina rivers are the main surface waterways of the area. Both rivers flow through the city and are tributaries of the San Juan River, which ends up in the El Cuchillo dam. However, the river channels of these rivers are dry most of the time and get water only during precipitation events.

Close to 40% of water supplied to MMA comes from shallow and deep boreholes located in three main wellfields: “Topo Chico,” “Area Metropolitana,” and “Buenos Aires.” Topo Chico wellfield is located to the north of Monterrey and is considered a fractured limestone aquifer. In this wellfield, 13 shallow wells have been drilled, 12 for public use and 1 for industrial use (CONAGUA 2015a). The stratigraphic column of this area is represented by a geological interval from Upper Jurassic to recent. The local Mesozoic sequence begins with gypsum, anhydrite, and limestone deposits. Continuously, deposits of sandstones, shales, and limestones can be found in a reducing environment of shallow waters. It is followed by limestones deposited in warm waters, which generated reef and perireef rocks constituted by an alternation of sandstones and shales (CONAGUA 2015a). The Area Metropolitana wellfield includes eight municipalities of the MMA and consists of a highly permeable aquifer in the alluvium-conglomerate formation. The wells located in the Monterrey valley are shallow wells (depth of wells ranges from 32 to 100 m) where smooth tubes with diameters of 12, 14, 18, and 20 in. have been installed. The geological column of this area covers weakly cemented conglomerate sediments with clay-sandy matrix, with thicknesses ranging from 1 to 30 m. Under these sediments, there are rocks of the Upper Cretaceous represented by shales and/or clay-limestones, although Middle Cretaceous limestones can also be found in wells located very near the mountain flanks of SMO. The strainer depth of wells located in the Monterrey valley ranges from 8 to 30 m.

The Buenos Aires wellfield is located to the west of MMA, inside of the mountain belts of SMO (700–1000 masl). Forty-two deep wells equipped with slotted pipes of 10 and 12 in. of diameter have been drilled in that area for public and urban use, in order to supply water to MMA (CONAGUA 2015b). The depth of these wells varies between 720 and 830 m; however, their strainer depths range between 23 and 100 m. Because this semi-confined aquifer is located at a higher altitude to the city and is very close to the recharge zones, it provides high-quality water with low cost of distribution. Thus, the Buenos Aires is the most important wellfield and contributes with 46% of the groundwater supplied to MMA (Masuch et al. 2002).

### Geology and hydrogeology

As mentioned above, the MMA is bordered on the southwest and south by SMO. This 2–3-km-thick Mesozoic belt consists of marine sedimentary material deposited over a complex basement that includes Meso and Neoproterozoic metamorphic rocks, Paleozoic schist/sedimentary, and Upper Triassic–Lower Jurassic red beds. The rock units of SMO include carbonates, evaporites, and siliciclastic material, whose deposition and facies distribution varied through time as a result of tectonics, fluctuations of the sea level, and detrital source variation (Velasco-Tapia et al. 2016). However, limestones and gypsum from Lower Cretaceous dominate the mineralogical composition. In the valley borders of the MMA, sedimentary rocks are composed of limestones and shale outcroppings, which also form gently hills inside the valley (Montalvo-Arrieta et al. 2008).

Toward the Santiago municipality, between Cerro La Silla and SMO, the lithology comprises limestones and lutites from the Upper Cretaceous. Nevertheless, inside the valley of Monterrey, there are three identified formations: (i) the Méndez Formation, a sequence of shale, calcareous shale, and stratified calcareous marl from the Upper Cretaceous, with a thickness of 1500–2800 m (Dávila-Pórcel 2011); (ii) the Reynosa Formation, composed by Tertiary conglomeratic sediments such as gravels, clays, and sands with traces of gypsiferous material and caliche deposits, with a maximum thickness of 60 m (CONAGUA 2015c); and (iii) alluvial sediments from Quaternary age, which were deposited during accumulation-erosion cyclic changes and are constituted by uncemented and unweathered cobble to small pebble gravel, gravelly sand, sand, and silts, locally cemented in modern washes by calcite cement (Montalvo-Arrieta et al. 2008). Figure 1 shows the geological map of the studied area.

## Materials and methods

### Sampling and analysis

The field and laboratory work consisted of sampling and a chemical analysis of groundwater from several wells that supply water to MMA. The field sampling campaigns were carried out in the years 2006 and 2012. Fourteen wells were sampled in November 2006 for chemical analyses. Similarly, 16 wells were sampled in a second campaign performed in April 2012 (which included eight wells sampled in the first campaign and eight new wells). Overall, 22 wells were sampled in both campaigns (Fig. 1). The wells sampled in the valley of Monterrey were codified with M, the wells sampled close to Santiago municipality were codified with S, while the wells sampled in Buenos Aires wellfield were categorized with the letter B. All the wells were equipped with pre-installed pumps. Auxiliary parameters (pH, temperature, and electrical conductivity) were determined onsite using pre-calibrated electrodes. Water samples were filtered in the field through 0.45-μm acetate cellulose membranes. Alkalinity was also measured onsite by acid titration until end point of pH 4.3 in filtered water samples. After filtering, the water samples were placed in low-density polyethylene sampling kits (three bottles of 250 ml for cations, anions, and trace element analyses), which were pre-washed and pre-rinsed several times prior to use. The samples for cation, silica, and trace element analyses were acidified with ultrapure HCl to pH < 2 in order to prevent major and trace element precipitation/adsorption during storage. All the water samples were stored at a constant temperature of 4 °C.

All the chemical analyses were performed in Activation Laboratories Ltd., Ancaster, Ontario, Canada. The concentrations of dissolved major cations (Na, K, Ca, Mg, and Si) were measured by inductively coupled plasma optical emission spectrometry (ICP-OES) using an Agilent Axial ICP Optical Emission Spectrometer. Major anions (Cl^−^, SO_4_^2−^, NO_3_^−^) were determined and quantified by ion chromatography in un-acidified samples using a Dionex DX-120 equipment. Samples with high Cl^−^ (>75 mg/L) and SO_4_^2−^ (>375 mg/L) concentrations were diluted in order to avoid oversaturation. Selected dissolved trace elements (As, Cd, Co, Cr, Cu, Mn, Mo, Pb, Se, U, and Zn) were measured by inductively coupled plasma mass spectrometry (ICP-MS) using a Perkin Elmer SCIEX ELAN 6000 ICP/MS equipment. During the trace element determinations, a blank and two water standards were run at the beginning and end of the analyses. Measurement uncertainties for all parameters were evaluated and controlled using regular laboratory duplicates of samples and verifying the precision/calibration of the instruments through regular runs of various primary standard solutions. The international geostandard SRM-1640 (trace elements in natural water—certified by the National Institute of Standards and Technology NIST) was measured during the analyses of groundwater samples to check the accuracy of the ICP-OES and ICP-MS methods. The accuracy of these analyses (defined as the systematic difference between the reference values and the measurements of the geostandard SRM-1640) was lower than 9% for major ions and trace elements. Table 1 shows the detection limits for major cation, anion, and trace element analyses reported by Activation Laboratories Ltd.

**Table 1:** Limits of detection reported for the analyses of major cations (ICP-OES), major anions (ion chromatography), and trace elements (ICP-MS) in groundwater samples collected during this study

	Na^+^ (mg/L)	K^+^ (mg/L)	Ca^2+^ (mg/L)	Mg^2+^ (mg/L)	Si (mg/L)	Cl^−^ (mg/L)	SO_4_^2−^ (mg/L)	NO_3_^−^ (mg/L)	Mn (μg/L)	Cr (μg/L)
Limit of detection	0.1	0.1	0.1	0.1	0.1	0.03	0.03	0.01	0.1	0.5
	Co (μg/L)	Cu (μg/L)	Zn (μg/L)	As (μg/L)	Se (μg/L)	Mo (μg/L)	Cd (μg/L)	Pb (μg/L)	U (μg/L)	
Limit of detection	0.005	0.2	0.5	0.03	0.2	0.1	0.01	0.01	0.001	

### Statistical analyses

The geochemical code PHREEQC (Parkhurst and Appelo 2013) and the dataset were used to evaluate the saturation index for the groundwater samples. Hierarchical cluster analysis (HCA) was applied to identify groups exhibiting similar water characteristics. This statistical method is a useful tool to organized water samples into classified groups (Mahlknecht et al. 2004). Thus, field parameters and major ions and trace element concentrations were selected and used in an HCA by means of Wards linkage rule. One-way ANOVA test was performed in order to establish significant differences among the groups defined by the HCA. A principal component analysis (PCA) was carried out in order to develop a primarily qualitative approach to analyzing the relationships among the measured variables. For geochemical data, an orthogonal method should be chosen for factor rotation during PCA (Reimann et al. 2002). Therefore, the varimax method was used. All the data were log-transformed and standardized (values minus mean divided by standard deviation) prior to application of multivariate statistical analyses. Indeed, this fact approximates normality and gives equal weight to all variables (Güler et al. 2002). Because several trace element concentrations were below the detection limit (DL) of the applied analytical method, the results of these values were expressed as DL/2 for statistical analysis purposes. The multivariate statistical analyses were performed using the computer software Statistica 7.0.

## Results

**Fig. 2:**
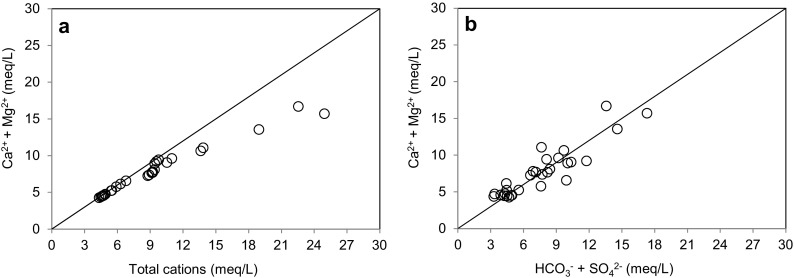
Scatters plots of **a**) Ca^2+^ + Mg^2+^ versus total cation concentrations and **b**) Ca^2+^ + Mg^2+^ versus HCO_3_^−^ + SO_4_^2−^ concentrations

The results of the analyzed variables in water samples collected in the years 2006 and 2012 in several wells that supply water to MMA are shown in Table 2. The electrical conductivity values are not presented in Table 2. However, this variable ranged between 400 and 2410 μS/cm, with an average of 880 ± 635 μS/cm. The pH values of groundwater varied between 6.77 and 7.88, indicating neutral to slightly alkaline water conditions within the studied area. The groundwater temperature changes according the flow path. Wells located in the valley of Monterrey and toward the northeast of Monterrey (samples codified with M) had water temperature values between 22.6 and 29.2 °C, with an average of 25.5 ± 1.6 °C. However, the wells located in higher areas close to recharge zones such as the Santiago municipality, foothills of SMO and the Buenos Aires wellfield, had colder groundwater, which showed temperature values between 18.9 and 24.7 °C, with an average of 21.1 ± 1.5 °C. On the other hand, the Si concentrations lie in the common range of 1–30 mg/L in natural waters (Hem 1989). The dominance of anions was in the order HCO_3_ > SO_4_ > Cl > NO_3_, while the concentrations of cations declined in the order Ca > Mg > Na > K for water samples taken in the Buenos Aires wellfield and several places located in the foothills of SMO and Ca > Na > Mg > K for water samples taken in the valley of Monterrey. In general, Zn was the most abundant of the measured trace elements in the water samples, whereas the concentrations of Cd and Co were the lowest. The concentrations of Na^+^, Cl^−^, SO_4_^2−^, NO_3_^−^, and all the selected trace elements showed a high variability, with standard deviations larger than the average values. Indeed, this fact can suggest a large spatial heterogeneity of groundwater chemistry in the studied area. The scatter diagram of (Ca^2+^ + Mg^2+^) versus total cation concentrations presented in Fig. 2a shows that the equilibrium ratios between both variables lie along the equiline. The ratio approximates to 1 at lower mineralization of water, indicating that these alkaline earth elements are the main cations present in groundwater. However, at higher mineralization, the data deviate from the equiline, suggesting the contribution of other cations such as Na^+^ and K^+^ to the groundwater chemistry. Additionally, the (Ca^2+^ + Mg^2+^) versus (HCO_3_^−^ + SO_4_^2−^) plot (Fig. 2b) shows that most points fall along the equiline, indicating that the abundance of these ions is mainly due to the weathering of carbonates (calcite and dolomite) and several salt rocks such as anhydrite and/or gypsum.

**Table 2:** Values of pH, temperature (°C), and major ion, Si, and trace element concentrations in all the groundwater samples collected along the studied area

Sample	pH	Temp	Na	K	Ca	Mg	Cl	HCO_3_	SO_4_^2^	NO_3_	Si	Mn	Cr	Co	Cu	Zn	As	Se	Mo	Cd	Pb	U
M1	7.60	26.4	1.74	0.43	78.9	3.74	2.45	270	12.5	1.17	3.0	19.5	< DL	< DL	7.50	11.6	0.22	0.6	0.5	0.02	0.59	0.61
M2	7.15	25.2	32.7	2.7	120	20.2	37.0	390	86.4	8.29	9.4	20.9	12.2	0.127	47.7	88.9	0.43	2.3	1.1	0.04	6.16	2.50
M3	6.96	25.1	29.0	2.06	129	20.1	35.4	378	107	10.6	10.0	33.1	1.0	0.091	4.3	25.5	0.47	3.3	1.1	0.05	0.65	2.10
M4	7.71	25.2	33.8	2.23	112	21.7	11.0	395	60.6	2.31	8.9	3.6	0.7	0.081	11.7	15.3	0.44	2.0	1.4	0.02	1.25	2.31
M5	6.95	28.0	67.6	1.74	181	19.5	131	342	197	20.5	10.6	4.7	< DL	< DL	3.0	184	0.86	5.4	3.3	0.64	1.52	2.06
M6	6.77	22.6	121	5.15	211	36.8	153	427	365	12.4	16.0	8.8	< DL	0.382	3.6	26.0	1.73	34.6	3.1	0.03	0.33	4.51
M7	7.65	24.4	34.1	2.32	121	20.1	35.8	321	90.6	8.71	11.6	0.3	9.5	0.096	0.7	128	0.96	2.7	2.1	0.01	0.47	2.43
M8	6.90	25.4	211	1.98	229	52.0	226	474	458	19.0	21.9	0.4	0.5	0.519	2.1	10.9	1.40	13.2	5.4	0.02	0.17	7.99
M9	6.94	25.4	133	2.94	254	48.8	189	300	416	15.7	20.6	0.4	1.0	0.284	1.6	73.7	2.11	32.6	3.2	0.08	0.17	5.52
M10	7.57	24.7	32.3	2.07	123	20.0	38.2	300	94.3	9.71	12.0	0.6	2.5	0.169	2.0	47.2	0.50	3.0	2.0	0.03	0.14	1.90
M11	7.35	24.3	33.6	2.56	109	22.0	42.5	300	83.0	9.19	10.9	0.2	3.4	0.177	1.0	16.4	0.47	2.1	2.8	< DL	0.31	2.06
M12	7.45	29.2	62.8	1.83	191	18.7	134	204	207	11.8	13.1	1.0	< DL	< DL	0.9	145	1.14	5.3	4.4	0.58	1.68	1.81
S1	7.04	21.6	5.03	0.66	123	5.13	6.96	537	54.1	3.41	7.5	1.4	< DL	< DL	2.0	165	0.11	0.9	0.2	< DL	0.31	0.51
S2	6.97	19.2	8.26	1.23	175	5.63	6.25	488	181	1.60	9.0	1.4	< DL	< DL	3.7	344	0.14	1.2	0.7	0.04	0.56	0.89
S3	7.05	19.2	11.5	0.67	169	5.71	10.1	390	176	1.50	9.9	15.8	< DL	0.023	1.2	142	0.09	1.0	0.5	0.02	0.28	1.00
S4	6.84	22.1	33.6	0.80	153	17.2	30.0	488	114	2.45	13.5	17.2	< DL	< DL	2.2	20.7	0.13	1.0	0.2	0.02	0.25	0.41
S5	7.20	21.0	5.54	0.69	97.6	4.51	3.98	305	27.2	1.77	7.4	0.5	< DL	< DL	2.4	9.7	0.23	0.9	0.2	< DL	0.49	0.50
S6	7.55	18.9	2.79	0.47	104	7.00	1.82	329	106	0.28	5.5	0.3	< DL	< DL	0.4	3.1	0.21	0.6	1.1	< DL	0.13	1.16
S7	7.50	20.6	2.58	0.53	79.4	6.91	2.02	256	36.0	0.57	4.6	0.2	< DL	< DL	0.5	2.3	0.23	0.6	0.9	< DL	0.14	0.92
S8	7.70	20.1	4.36	0.76	82.4	13.5	1.91	216	44.7	0.73	5.5	0.3	< DL	0.032	0.3	2.9	0.41	0.9	2.3	0.01	0.06	1.24
S9	7.88	20.5	3.34	0.53	109	8.43	1.64	135	107	0.33	6.7	< DL	< DL	0.029	< DL	3.9	0.38	1.1	2.1	0.03	0.07	1.24
S10	7.79	20.4	3.22	0.58	81.1	8.52	1.71	216	37.8	0.68	5.3	0.2	< DL	0.031	0.3	1.3	0.29	1.0	1.8	0.02	0.06	0.96
S11	7.78	19.9	3.12	0.57	77.5	7.71	1.74	255	34.5	0.68	5.4	0.1	< DL	0.030	0.2	1.4	0.33	1.0	1.6	0.01	0.07	0.87
S12	7.27	23.2	7.24	0.85	181	4.94	4.26	252	192	1.56	9.6	0.5	< DL	0.091	1.6	12.6	0.27	1.3	1.7	0.04	0.14	0.89
S13	7.05	24.7	31.5	0.54	165	16.6	32.0	414	116	1.39	14.8	24.7	< DL	0.151	0.3	4.1	0.23	4.9	0.5	0.01	0.04	0.44
B1	7.54	22.1	3.03	0.72	72.3	13.4	2.09	190	12.5	1.0	4.3	1.7	< DL	< DL	3.3	9.1	0.31	0.8	1.6	0.02	0.38	1.24
B2	7.22	22.4	3.90	0.81	72.2	11.8	2.79	195	34.9	1.36	5.5	0.4	< DL	< DL	1.2	4.2	0.60	1.1	4.5	0.01	0.24	1.62
B3	7.58	21.9	2.26	0.66	68.8	11.3	1.66	180	16.4	0.92	4.4	0.2	< DL	< DL	1.0	5.0	1.51	1.0	2.2	0.01	0.39	0.80
B4	7.56	21.6	2.75	0.70	72.1	10.4	1.90	250	20.2	0.95	4.5	0.5	< DL	< DL	2.2	8.6	0.64	0.8	2.5	0.03	0.34	1.13
B5	7.61	21.3	3.09	0.67	71.5	10.8	1.84	234	19.9	1.0	4.7	1.1	< DL	0.057	9.5	17.6	0.37	0.6	2.4	0.04	0.49	1.12
Mean	7.34	22.9	31.0	1.35	127	15.8	38.3	314	117	5.05	9.20	5.3	1.21	0.08	3.95	51	0.57	4.26	1.9	0.06	0.60	1.76
SD	0.32	2.6	46.7	1.05	51	11.8	60.8	103	115	5.87	4.67	8.8	2.69	0.12	8.55	77	0.51	8.22	1.3	0.15	1.11	1.61

The concentrations of major ions (Na^+^, K^+^, Ca^2+^, Mg^2+^, Cl^−^, HCO_3_^−^, SO_4_^2−^, NO_3_^−^) and Si are in mg/L. Trace element (Mn, Cr, Co, Cu, Zn, As, Se, Mo, Cd, Pb, and U) concentrations are in μg/L

*< DL* value lower than the detection limit of the analytical technique, *SD* standard deviation

Regarding trace element abundances, the mean concentration values of these in groundwater of the studied area (with the exception of Pb) were lower than the mean concentrations of trace elements in groundwater supplied to other megacities such as Tokyo (Mn 82 μg/L, Cu 3.7 μg/L, Se 4.7 μg/L, Cr 1.8 μg/L, As 1.8 μg/L, and Cd 0.1 μg/L), mainly because groundwater in that region is in contact with volcanic ash soils (which can be rich in trace elements) and unconsolidated layers of silt, sand, and gravel (Thuyet et al. 2016). However, the mean trace element contents in groundwater supplied to MMA were higher than those found in groundwater supplied to heavily urbanized areas of Hong Kong (Mn 2.72 μg/L, Cu 1.16 μg/L, Zn 40.8 μg/L, Se 0.38 μg/L, Cr 0.71 μg/L, Co 0.02 μg/L, As 0.42 μg/L, Mo 0.29 μg/L, and Cd 0.07 μg/L), a region in which the geology is dominated by acidic volcanic rocks and granite bedrocks (Leung and Jiao 2006).

## Discussion

### Principal component analysis (PCA)

PCA is a statistical method that reduces the dimensionality of the data while retaining most of its variation. It transforms all data into several principal components (PCs), which express common properties of the variables without losing information of the original data (Ringner 2008). In this study, PCA captures the important features inherent in the variability of major ions and dissolved trace elements in groundwater and helps to recognize patterns controlling the content of these ions and elements. Table 3 shows the results of the PCA applied to the data of physicochemical variables, major ions, and dissolved trace elements in groundwater supplied to the MMA. Four important components were significant. The first (PC1), which describes most of the sample variance (44%), has high negative loads for the major ions (Na^+^, K^+^, Ca^2+^, Mg^2+^, Cl^−^, SO_4_^2−^, NO_3_^−^), Si, and several trace elements such as Co, Zn, and Se. This component is related to the weathering of carbonates (calcite and dolomite), silicates, and several salt rocks such as anhydrite (CaSO_4_), gypsum (CaSO_4_·2H_2_O), halite (NaCl), and sylvite (KCl). Nitrate (NO_3_^−^) can also be derived from geological sources, mainly in arid and semi-arid regions due to the occurrence of nitratine (NaNO_3_) and nitre (KNO_3_) in caliche deposits (Holloway and Dahlgren 2002). Thus, this fact indicates that the weathering process provides an overwhelming proportion of the dissolved load and controls the major ion chemistry in groundwater of the area. Although bicarbonate (HCO_3_^−^) shows a positive relationship with the other major ions in the first component, this anion is more related with the second component (Table 3). This could suggest that most of major ions in highly mineralized waters are originated by the weathering of evaporites, because the weathering of carbonates, silicates, and K-feldspar implicates the release of bicarbonate ions to groundwater. Similarly, the high association of Co, Zn, and Se with major ions in the first component can suggest that these elements are released to groundwater during the rock weathering process.

**Table 3:** Results of the principal component analysis (PCA)

Variables	Components			
	PC1	PC2	PC3	PC4
Temp	−0.59	0.22	−0.38	0.15
pH	0.63	0.51	−0.19	−0.10
Na^+^	**−0.97**	−0.02	0.16	−0.02
K^+^	**−0.90**	0.12	−0.16	−0.23
Ca^2+^	**−0.80**	−0.25	0.41	0.24
Mg^2+^	**−0.82**	0.39	0.01	−0.15
Cl^−^	**−0.92**	−0.06	0.00	0.07
SO_4_^2−^	**−0.76**	−0.08	0.49	0.14
NO_3_^−^	**−0.89**	−0.03	−0.22	−0.01
HCO_3_^−^	−0.49	**−0.69**	0.21	−0.28
Si	**−0.88**	−0.08	0.42	−0.03
As	−0.47	**0.72**	−0.25	0.10
Cd	−0.45	−0.04	−0.36	**0.71**
Co	**−0.58**	0.29	0.33	−0.42
Cr	−0.49	0.11	−0.25	**−0.65**
Cu	−0.39	−0.43	**−0.67**	−0.15
Mn	−0.37	**−0.68**	−0.20	0.08
Mo	−0.24	**0.86**	−0.21	0.15
Pb	−0.39	−0.30	**−0.82**	0.00
Se	**−0.90**	0.20	0.19	0.09
U	−0.52	**0.62**	−0.23	−0.10
Zn	**−0.61**	−0.47	−0.23	0.13
% of variance	44.0	16.9	12.0	6.7

The second component (PC2) describes 16.9% of the sample variance and has high positive loads for As, Mo, and U and high negative loads for HCO_3_^−^ and Mn. It appears that this component is associated to redox processes controlling the solubility of As, Mo, U, and Mn in groundwater. The third component describes the 12% of the variance, and it is related with the mobility of Cu and Pb in groundwater. The fourth component (PC4) describes 6.7% of the variance and shows a negative relationship between Cr and Cd. However, because most samples showed Cr concentrations below the detection limit of the reported method (<0.5 μg/L), no discussion about this negative relationship was made in order to avoid speculations.

### Hierarchical cluster analysis (HCA)

**Fig. 3:**
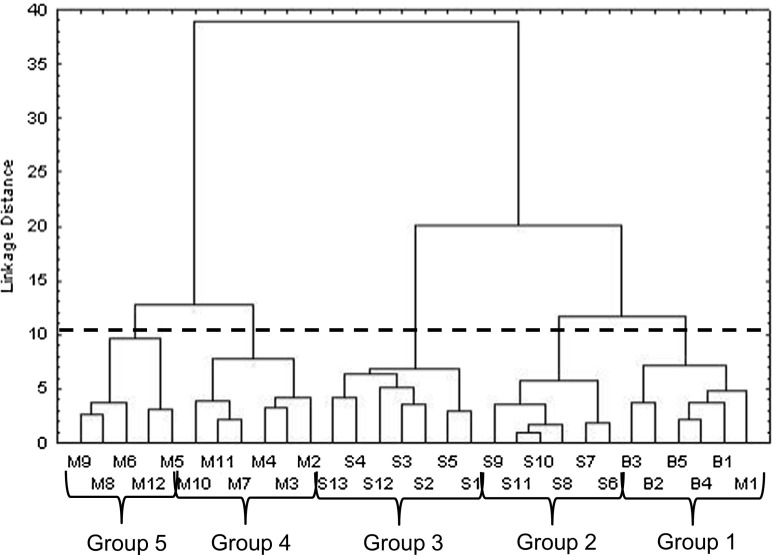
Dendrogram with imaginary horizontal line (phenon line) to define five groups of water samples based on a hierarchical cluster analysis of cases using log-transformed and standardized dataset

Cluster analyses of cases were performed on the analytical data to detect similarities and differences among water samples taken in different wells. The resulting dendrogram suggest five groups at the phenon line: (i) water samples taken in the Buenos Aires wellfield; (ii) water samples taken in the foothills of the SMO, toward Santiago municipality; (iii) water samples taken in the Santiago municipality; (iv) water samples taken in the MMA aquifer; and (v) water samples taken in the Topo Chico wellfield (north of MMA) and in the northeast of MMA. However, although the sample M1 was taken in the valley of Monterrey, this water sample was associated with the group 1 of waters. This can be due to the fact that this well is located toward the foothills of La Silla mountain, which has the same lithology that the SMO mountain belts. Figure 3 shows the dendrogram obtained when HCA was applied to analytical data using the Ward’s method.

Table 4 shows the mean and standard deviation values of the five groups according to HCA. Also, this table shows the results of the one-way ANOVA test, which was applied between variables of each group in order to establish significant differences among groups of waters.

**Table 4:** Average and standard deviation (SD) values of the chemical parameters of the five groups according to hierarchical cluster analysis (HCA)

	Cluster				
Groups	1	2	3	4	5
Variables	Average ± SD	Average ± SD	Average ± SD	Average ± SD	Average ± SD
pH	7.51 ± 0.15b	7.70 ± 0.15b	7.06 ± 0.14a	7.40 ± 0.30b	7.00 ± 0.26a
Na^+^ (mg/L)	2.80 ± 0.74d	3.24 ± 0.62d	14.7 ± 12.4c	32.6 ± 1.9b	119 ± 60a
K^+^ (mg/L)	0.67 ± 0.13b	0.57 ± 0.10b	0.78 ± 0.22b	2.32 ± 0.26a	2.73 ± 1.44a
Ca^2+^ (mg/L)	72.6 ± 3.3e	88.9 ± 13.8d	152 ± 31c	119 ± 7b	213 ± 29a
Mg^2+^ (mg/L)	10.2 ± 3.4c	8.7 ± 2.5c	8.5 ± 5.7c	20.7 ± 0.9b	35.2 ± 15.7a
Cl^−^ (mg/L)	2.12 ± 0.42c,d	1.81 ± 0.14d	13.4 ± 12.2c	33.3 ± 11.2b	167 ± 40a
SO_4_^2−^ (mg/L)	19.4 ± 8.3c	61 ± 35b	123 ± 65b	87 ± 15b	329 ± 120a
NO_3_^−^ (mg/L)	1.07 ± 0.17e	0.55 ± 0.19d	1.95 ± 0.73c	8.14 ± 2.96b	15.9 ± 3.9a
HCO_3_^−^ (mg/L)	220 ± 37c	235 ± 64b,c	411 ± 104a	347 ± 45a	349 ± 106a,b
Si (mg/L)	4.40 ± 0.81d	5.50 ± 0.68c	10.2 ± 2.9b	10.5 ± 1.2b	16.4 ± 4.8a
TDS (mg/L)	345 ± 34c	405 ± 76c	735 ± 144b	661 ± 43b	1247 ± 341a
As (μg/L)	0.61 ± 0.47b,c,d	0.31 ± 0.08d	0.17 ± 0.07c	0.55 ± 0.20b	1.45 ± 0.49a
Cd (μg/L)	0.02 ± 0.01a	0.01 ± 0.01a	0.02 ± 0.02a	0.03 ± 0.02a	0.27 ± 0.31a
Co (μg/L)	0.012 ± 0.022a	0.021 ± 0.014a	0.039 ± 0.059a	0.12 ± 0.04b	0.24 ± 0.23a
Cr (μg/L)	<0.5b	<0.5b	<0.5b	4.9 ± 4.8a	0.45 ± 0.33a
Cu (μg/L)	4.1 ± 3.5a	0.3 ± 0.1b	1.9 ± 1.1a	11.2 ± 18.3a,b	2.2 ± 1.1a
Mn (μg/L)	3.90 ± 7.66a	0.19 ± 0.10a	8.79 ± 10.16a	9.78 ± 13.94a	3.06 ± 3.68a
Mo (μg/L)	2.3 ± 1.3a,b	1.6 ± 0.6b	0.6 ± 0.5c	1.8 ± 0.7b	3.9 ± 1.0a
Pb (μg/L)	0.41 ± 0.12a	0.09 ± 0.04b	0.30 ± 0.18a,b	1.50 ± 2.32a,b	0.77 ± 0.76a,b
Se (μg/L)	0.8 ± 0.2c	0.9 ± 0.2c	1.6 ± 1.5c,b	2.6 ± 0.5b	18.2 ± 14.4a
U (μg/L)	1.1 ± 0.4c	1.1 ± 0.2c	0.7 ± 0.3b	2.2 ± 0.2a	4.4 ± 2.6a
Zn (μg/L)	9.35 ± 4.88c	2.48 ± 1.02b	100 ± 127a,b,c	54 ± 46a	88 ± 75a

Different letters between groups for a particular variable denote significant differences at *p* < 0.05 according to one-way ANOVA test

### Chemistry of major ions

**Fig. 4:**
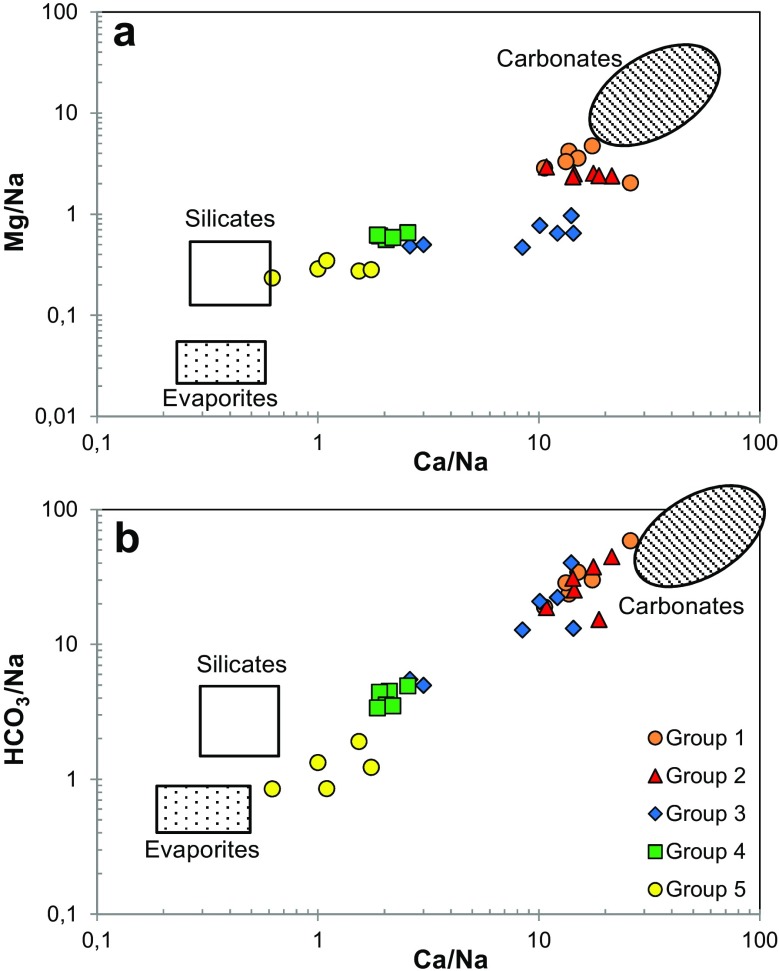
Mixing diagrams as molar ratios of **a**) Mg/Na versus Ca/Na and **b**) HCO_3_^−^/Na versus Ca/Na for the five groups of water defined by the HCA. The carbonate, evaporite, and silicate rock fields are from Gaillardet et al. (1999)

Chemical composition of groundwater depends principally on the local geology because water-rock interaction is the most important factor that influences the water’s chemistry. Figure 4 shows a log-log scaled binary diagrams with Na-normalized molar ratios (Ca/Na versus Mg/Na and Ca/Na versus HCO_3_/Na) together with the potential rock weathering “end-members,” which are useful to distinguish waters interacting with carbonates, silicates, and evaporite rocks (Gaillardet et al. 1999). Molar ratios of groups of water 1 and 2 almost lie within the carbonate field, indicating that these groups of waters have had a strong interaction with calcite and dolomite, which are the dominant rocks in the SMO. The waters of group 3, which were taken in the Santiago municipality, show a strong interaction with carbonate rocks, although they also show a higher interaction with silicates and evaporite rocks than the previous two groups. On the other hand, the ratios of the waters taken in the valley of Monterrey (group 4) follow evaporite-carbonate-silicate mixing, whereas the group 5 of waters tends to be located toward the evaporite end-member, suggesting a strong water interaction with salt rocks such as gypsum, anhydrite, halite, and sylvite.

The results from HCA and one-way ANOVA (Table 3) indicate that groundwater coming from Buenos Aires wellfield and foothills of SMO (groups 1 and 2) has low concentrations of major ions. These two groups had significantly lower concentrations of Na^+^, K^+^, Ca^2+^, Mg^2+^, Cl^−^, NO_3_^−^, Si, and total dissolved solids (TDS) than the groups 4 and 5. Both groups of samples, which were taken in recharge zones, are of Ca-HCO_3_^−^ water type and represent the least mineralized waters of the all groups of the studied area, reflecting a short groundwater flow path. Also, owing to the weathering of carbonates in the SMO, both groups are saturated to oversaturated with respect to aragonite, calcite and, in several cases, in equilibrium with respect to dolomite. The differences among these two groups lie in the concentrations of Ca^2+^, SO_4_^2−^, NO_3_^−^, and Si, which were statistically different at *p* value lower than 0.05 (Table 3).

The waters of group 3, which were sampled in wells of the Santiago municipality, can also be classified as Ca-HCO_3_^−^ type. According with the topographic flow paths shown in Fig. 1, waters of the group 3 come from recharge areas located in the foothills of the SMO in front of Santiago (group 2 of waters) and flows through siliciclastic rocks, limestones, and lutites from the Upper Cretaceous located in Santiago. Owing to the increasing groundwater transit time, the rock weathering process continues, producing a significant increase in the concentrations of Na^+^, Ca^2+^, Cl^−^, NO_3_^−^, HCO_3_^−^, and Si and in waters of group 3 with regard to the waters of group 2 (Table 3).

Table 3 indicates that waters of the group 4 have significantly higher concentrations of Na^+^, K^+^, Mg^2+^, Cl^−^, and NO_3_^−^ than the first three groups, and higher concentrations of Si and Ca^2+^ than the first two groups. Although this aquifer has a high permeability, Fig. 4 shows that these waters have strongly interacted with carbonates, silicates, and evaporite rocks, probably due to the fact that they are coming from the SMO and flow through the alluvial sediments and calcareous shales of the Mendez Formation inside the valley of Monterrey. These transition zone waters are oversaturated with respect to aragonite, calcite, and dolomite, and they can be classified as Ca-HCO_3_-SO_4_ type.

Finally, group 5 is a Ca-SO_4_ water type. The waters of this group are also oversaturated with respect to aragonite, calcite, and dolomite, and they represent the most mineralized water group of the studied zone. Although these waters are not saturated with respect to any evaporitic phase, this group tends to be located toward the evaporite “endmember” (Fig. 3), because these waters show the lowest HCO_3_/Na and Ca/Na molar ratios and they have the significantly highest concentrations of Na^+^, Ca^2+^, Cl^−^, SO_4_^2−^, and NO_3_^−^ of all groups. Indeed, this fact suggests a strong interaction with halite, sylvite, gypsum, and anhydrite. Although elevated nitrogen concentrations in several water bodies have been attributed to weathering of bedrock nitrogen (Holloway and Dahlgren 2002), the NO_3_^−^ content in the group 5 of waters was above the threshold value (3 mg/L) for anthropogenic influence (Pastén-Zapata et al. 2014), indicating the existence of other NO_3_^−^ source. Thus, these increased NO_3_^−^ concentrations can be attributed to infiltration of surface contaminated waters coming from the MMA (sewage waters, leachate from municipal landfills and sanitary sewers, septic deposits, etc.).

Overall, the ionic composition of the groundwater of the studied area is in agreement with the topographic flow path: groundwater flows from recharge areas located in the foothills of SMO toward the valley of Monterrey and interacts with alluvial sediments and shales of the Mendez Formation. Then, groundwater moves away to the northeast and the weathering of rocks goes on, increasing the ionic composition of waters. Also, toward the northeast of the MMA, groundwater interacts with the Reynosa Conglomerate. Therefore, the weathering of carbonates, clays, and gypsiferous materials may significantly increase the concentrations of Na^+^, K^+^, Ca^2+^, Mg^2+^, SO_4_^2−^, and Si in waters (Mora et al. 2017).

### Chemistry of trace elements

The composition of trace elements in groundwater is determined by mineral dissolution and several geochemical processes. Trace elements can be mainly contained in silicates, but also, they can be associated to carbonate and evaporite minerals (Dean 1978; Fusswinkel et al. 2013). However, trace element concentrations in groundwater are controlled by several processes such as surface sorption/desorption, ion exchange, precipitation/dissolution, chelation, and microbial removal/input (Leung and Jiao 2006). In groundwater supplied to MMA, the positive relationships showed by Co, Se, and Zn with the major ions shown in PC1 (Table 3) could indicate that these trace elements are mobilized during the rock weathering and they remain dissolved during the transport process. This behavior is in agreement with tracer experiments performed in groundwater, in which several elements such as Co, Se, and Zn showed a high mobility under natural hydrogeological conditions (Müller 2000).

Table 3 also indicates that PC2 has high positive loads for the elements As, Mo, and U and a high negative load for Mn. This fact seems to be related with the redox conditions in groundwater of the studied area. In aquatic systems, Mo has shown a conservative behavior under aerobic conditions (oxidizing conditions) due to the formation of the MoO_4_^2−^ ion, which has a high mobility. However, under anoxic condition, the solubility of Mo decrease gradually due to the formation of the ion MoS_4_^2−^, which may sediment with particulate material and/or Fe-oxyhydroxides (Pizarro et al. 2014). Similarly, As tends to be more soluble under oxidizing conditions. Under those conditions, As forms the oxyanion arsenate (AsO_4_^3−^), which shows a moderate solubility, whereas under reducing conditions, As forms sulfides of very low solubility (Weiner 2007). On the other hand, Mn exhibits an opposite behavior than that shown by As and Mo. In oxygen-depleted groundwater, soluble Mn exists in the form of Mn^2+^ (Thuyet et al. 2016), which also shows a moderate solubility. However, alkaline and oxidizing conditions promote the oxidation of soluble Mn^2+^ to insoluble Mn-oxyhydroxides (such as Mn_2_O_3_, MnOOH, and MnO_x_) (Tebo et al. 2005).

The PCA carried out in groundwater of the MMA indicates that PC2 has a high positive load for U. Also U concentrations show a positive relationship with the major ions in PC1. Therefore, the behavior of U in groundwater of the studied area can be explained as follows: U has been considered a high mobile element in natural waters (Gaillardet et al. 2003), and its content in groundwater may be derived naturally from leaching of local rocks and soils (Nriagu et al. 2012). Indeed, this fact might explain the positive correlation with major ions. Conversely, U(VI) is much more soluble than U(IV) and may migrate as aqueous species in the environment (Arnold et al. 2011; Ribeiro et al. 2015). Thus, PC2 explains the more solubility of oxidized species of U, As, and Mo in groundwater of the MMA.

The PC3 indicates a positive relationship between Cu and Pb. Also, Table 4 indicates that there are few significant differences between the five groups of waters for Pb and Cu concentrations. These facts could suggest that Pb and Cu have a low mobility in groundwater and that the solubility of both elements is controlled by the same geochemical processes. Similar results have been found in groundwater supplied to western Tokyo, which have indicated that Pb and Cu are the less mobile elements in these waters (Thuyet et al. 2016). Furthermore, Chen et al. (2007) have found significant correlations between Pb and Cu in groundwater from coastal area in Shenzhen, China. Thus, the low concentrations and the relative low mobility of Pb and Cu in groundwater could be due to their high capacity to be adsorbed onto mineral surfaces, oxyhydroxides, clays, and organic and inorganic sediments (Leung and Jiao 2006; Thuyet et al. 2016).

### Comparison of water quality data with national and international guidelines

According to international guidelines for drinking water quality (WHO 2011) and the Mexican regulations for water quality (NOM-127 1994), the determined parameters (major ions and trace elements) in the first four groups of waters have concentrations that do not pose any risk to human health. However, all the water samples of group 5 showed NO_3_^−^ concentrations much higher (mean of 15.9 mg/L of NO_3_^−^-N) than the maximum level allowed by the Mexican normative (10 mg/L of NO_3_^−^-N) and the guideline value (11 mg/L of NO_3_^−^-N) proposed by WHO (2011) in order to protect bottle-fed infants against methemoglobinemia formation. Indeed, this fact indicates that the consumption of highly mineralized waters of group five is not recommended unless the high NO_3_^−^ concentrations are removed from them. In addition, one water sample of this group have Na^+^ levels above the national guidelines (200 mg/L), whereas three water samples of the same group have TDS and SO_4_^2−^ concentrations higher than the maximum values proposed by the Mexican normative (1000 mg/L for TDS and 400 mg/L for SO_4_^2−^). Conversely, the international guidelines for drinking water established by the World Health Organization (WHO 2011) indicates that reliable data on possible human health effects associated with the ingestion of water with high concentrations of Na, SO_4_^2−^, and TDS are not available, and no health-based guidelines values are proposed for these chemical parameters. However, these international guidelines establish that levels of Na and TDS higher than 200 and 1200 mg/L (respectively) in drinking water may give rise to unacceptable taste to consumers, whereas SO_4_^2−^ concentrations higher than 500 mg/L might cause a laxative effect on humans.

Our results also indicates that groundwater supplied to MMA have concentration values of the selected trace elements lower than the guideline values (As 10 μg/L, Cd 3 μg/L, Cr 50 μg/L, Cu 2000 μg/L, Mn 400 μg/L, Mo 70 μg/L, Pb 10 μg/L, Se 40 μg/L, and U 30 μg/L) proposed by WHO (2011) and by the Mexican normative (NOM-127 1994) (maximum permissible values of trace elements in waters indicated by the Mexican regulations for water quality are similar or higher than those proposed by WHO). However, several water samples of group five showed Se concentrations close to the provisional guideline value (40 μg/L) recently designated by WHO (2011). The high concentrations of Se in this Ca-SO_4_ water type can be related to the interaction of water with gypsiferous materials, because Se in groundwater is often in association with sulfur-containing minerals (WHO 2008).

## Conclusions

This paper reports the main chemical properties of the groundwater supplied to the MMA population. The HCA applied to chemical data of groundwater indicates that there are five groups of waters. The first two groups represent recharge waters of Ca-HCO_3_ type from the SMO. These are the least mineralized waters of the studied area and the differences between them lie in the concentrations of Ca^2+^, SO_4_^2−^, NO_3_^−^, and Si. Waters of group 3 are also of Ca-HCO_3_ type. These waters are coming from SMO and flow through lutites and limestones in the Santiago municipality. Transition zone waters of group 4 are of Ca-HCO_3_-SO_4_ type and flow through the alluvial sediments and calcareous shales located inside the valley of Monterrey. The waters of group 5 are of Ca-SO_4_ type and flow through conglomerates and alluvial sediments of the north and northwest of the MMA. Indeed, the ionic composition of water depends on the geology of the zone and the transit time of groundwater. Major ion composition and the content of Si, Co, Se, and Zn in groundwater are mainly controlled by rock weathering processes. The trace elements As, Mo, Mn, and U are grouped in PC2 because they are redox-sensitive elements, whereas other trace elements such as Pb and Cu seem to be the least mobile selected elements in groundwater of the studied area.

The groundwater coming from the SMO, the Santiago municipality and the Monterrey valley (first four groups of waters) are waters of high quality. However, the highest mineralized waters of group 5 have SO_4_^2−^ and TDS concentrations higher than the maximum levels allowed by the Mexican regulations for water quality. Also, all the water samples of group 5 have NO_3_^−^ concentrations higher than the guideline values proposed by both the Mexican normative and the World Health Organization in order to prevent formation of methemoglobinemia or “blue baby” syndrome in infants. Indeed, this indicates that the consumption of groundwater coming from the wells located to the north (Topo Chico wellfield) and northwest of the Monterrey valley is not recommended unless the high concentrations of NO_3_^−^, SO_4_^2−^ and TDS are removed from them.
